# The Association Between Persistent White-Matter Abnormalities and Repeat Injury After Sport-Related Concussion

**DOI:** 10.3389/fneur.2019.01345

**Published:** 2020-01-21

**Authors:** Benjamin L. Brett, Yu-Chien Wu, Sourajit M. Mustafi, Andrew J. Saykin, Kevin M. Koch, Andrew S. Nencka, Christopher C. Giza, Joshua Goldman, Kevin M. Guskiewicz, Jason P. Mihalik, Stefan M. Duma, Steven P. Broglio, Thomas W. McAllister, Michael A. McCrea, Timothy B. Meier

**Affiliations:** ^1^Department of Neurosurgery, Medical College of Wisconsin, Milwaukee, WI, United States; ^2^Department of Neurology, Medical College of Wisconsin, Milwaukee, WI, United States; ^3^Department of Radiology and Imaging Sciences, Indiana University School of Medicine, Indianapolis, IN, United States; ^4^Department of Radiology, Medical College of Wisconsin, Milwaukee, WI, United States; ^5^Department of Neurosurgery, David Geffen School of Medicine at University of California, Los Angeles, Los Angeles, CA, United States; ^6^Division of Sports Medicine, Departments of Family Medicine and Orthopedics, University of California, Los Angeles, Los Angeles, CA, United States; ^7^Department of Exercise and Sport Science, Matthew Gfeller Sport-Related Traumatic Brain Injury Research Center, University of North Carolina at Chapel Hill, Chapel Hill, NC, United States; ^8^School of Biomedical Engineering and Sciences, Wake-Forest and Virginia Tech University, Blacksburg, VA, United States; ^9^Michigan Concussion Center, School of Kinesiology, University of Michigan, Ann Arbor, MI, United States; ^10^Department of Psychiatry, Indiana University School of Medicine, Bloomington, IN, United States; ^11^Department of Biomedical Engineering, Medical College of Wisconsin, Milwaukee, WI, United States; ^12^Department of Cell Biology, Neurobiology and Anatomy, Medical College of Wisconsin, Milwaukee, WI, United States

**Keywords:** sport-related concussion, mTBI, diffusion tensor imaging, CARE, head injury, white matter

## Abstract

**Objective:** A recent systematic review determined that the physiological effects of concussion may persist beyond clinical recovery. Preclinical models suggest that ongoing physiological effects are accompanied by increased cerebral vulnerability that is associated with risk for subsequent, more severe injury. This study examined the association between signal alterations on diffusion tensor imaging following clinical recovery of sport-related concussion in athletes with and without a subsequent second concussion.

**Methods:** Average mean diffusivity (MD) was calculated in a region of interest (ROI) in which concussed athletes (*n* = 82) showed significantly elevated MD acutely after injury (<48 h), at an asymptomatic time point, 7 days post-return to play (RTP), and 6 months relative to controls (*n* = 69). The relationship between MD in the identified ROI and likelihood of sustaining a subsequent concussion over a 1-year period was examined with a binary logistic regression (re-injured, yes/no).

**Results:** Eleven of 82 concussed athletes (13.4%) sustained a second concussion within 12 months of initial injury. Mean MD at 7 days post-RTP was significantly higher in those athletes who went on to sustain a repeat concussion within 1 year of initial injury than those who did not (*p* = 0.048; *d* = 0.75). In this underpowered sample, the relationship between MD at 7 days post-RTP and likelihood of sustaining a secondary injury approached significance [χ^2^_(1)_ = 4.17, *p* = 0.057; *B* = 0.03, SE = 0.017; OR = 1.03, CI = 0.99, 1.07].

**Conclusions:** These preliminary findings raise the hypothesis that persistent signal abnormalities in diffusion imaging metrics at RTP following concussion may be predictive of a repeat concussion. This may reflect a window of cerebral vulnerability or increased susceptibility following concussion, though understanding the clinical significance of these findings requires further study.

## Introduction

A recent systematic review concluded that the physiological effects of concussion may persist beyond observed clinical recovery, typically based on self-reported symptoms, neurocognitive testing, and postural stability measures (1). Measurement of potential persistent physiological effects has largely used diffusion tensor imaging (DTI), an image technique to measure white-matter integrity derived from properties of water molecule diffusion, due to its sensitivity to detect abnormalities acutely following injury and its ability to track differences across time points in clinical recovery (1–4). Specifically, differences between concussed and non-injured controls have been commonly reported for different DTI metrics, including fractional anisotropy (FA, anisotropy of water diffusion) and mean diffusivity [MD; measure of average diffusion within tissue, regardless of direction; (4–7)], acutely and throughout injury recovery.

The observed direction of signal differences on DTI following sport-related concussion, however, has varied across studies (e.g., higher vs. lower signal following injury). For example, Lancaster et al. (7) observed diffuse decreased MD in recently concussed athletes as compared to controls acutely following injury (<24 h). Decreased MD has been observed by other similar studies comparing athletes to controls acutely following injury (6, 8). In contrast, Mustafi et al. (4) observed higher MD among acutely concussed athletes (<48 h), as compared to controls in select frontal and subfrontal white matter tracts (corpus callosum, the anterior and posterior corona radiata, and the superior longitudinal fasciculus). Elevations in MD in the bilateral longitudinal fasiculi and left corona radiata have also been observed acute among 26 recently concussed athletes (mean of 4 days since injury), as compared to matched controls (5). The reason for conflicting diffusion direction (increased vs. decreased anisotropy) acutely/subacutely after sport-related concussion across studies is not entirely clear, though one study examining potential study-related factors (e.g., participant age, publication year, voxel-based vs. ROI analysis method) observed a significant effect of the total diffusion-weighted images utilized within a particular study [i.e., great number of diffusion-weighted images associated with greater likelihood of reporting increased anisotropic diffusion; (9)].

Regardless of the observed direction, studies have shown that early diffusion metrics correlate with traditional clinical measures following concussion [e.g., inventory of concussion-related symptoms, inventory of psychological symptoms of distress, and time to RTP; (10)]. Other studies have shown that persistent signal alterations remain even after symptom resolution and RTP (2, 7, 11). However, the clinical significance and practical implications of *persistent signal alterations* in diffusion metrics following concussion are unknown. One hypothesis, based on pre-clinical and limited clinical studies, is that athletes cleared to return to play (RTP) before complete physiological recovery of an initial injury are at heightened risk for a subsequent, possibly more severe injury (12–14). Considered together, it has been suggested that persistent signal alterations on DTI reflect a period in which the brain has not yet fully recovered, or an ongoing window of cerebral vulnerability (14). The notion of an ongoing window of cerebral vulnerability is supported by findings from one of the earlier studies of NCAA athletes, which observed that 92.0% of repeat concussions within the same season occurred within 10 days of initial injury (15). This hypothesis that persistent diffusion signal following concussion is associated with repeat concussion has not been directly tested in the context of concussion due to decreased base rates of repeat concussion within the same year (16).

The National Collegiate Athletic Association (NCAA) and the US Department of Defense (DoD) Grand Alliance Concussion Assessment, Research and Education (CARE) Consortium is a multi-center study conducted over multiple years that provides a unique opportunity to address this hypothesis. Recent work from the CARE Consortium observed persistent elevations in MD in concussed athletes in the corpus callosum acutely following injury (i.e., 24–48 h), as well as at three additional time points after clinical recovery: following resolution of symptoms (asymptomatic), 7 days following unrestricted RTP (post-RTP), and 6 months post-injury (10). The current study investigated whether persistent DTI signal (i.e., elevations in MD) in these previously identified regions were different among athletes with and without a subsequent re-injury within the following 1-year period.

## Methods

### Participants and Procedures

Athletes were recruited between August 2014 and January 2018. This study was approved by the Medical College of Wisconsin Institutional Review Board and the Human Research Protection Office (HRPO) and written informed consent was obtained from all participants. Baseline clinical testing was performed during the preseason (17). Eighty-two athletes sustained concussions and completed the post-injury neuroimaging protocol.

Neuroimaging and clinical data were collected at the 24–48 h, asymptomatic, post-RTP, and 6-month visits (10). Athletes were followed over multiple years of sport participation, which allowed tracking of re-injury incidence. Data from the 6-month follow-up visit were not analyzed due to 4 of 11 re-injured athletes experiencing their second injury before this time. Athletes with a neuroimaging data available from at least one of the three above visits were included within the analyses. Neuroimaging data from each of the four visits were available as follows: 24–48 h (*n* = 53; *n* = 10 of the re-injured group), asymptomatic (*n* = 52; *n* = 10 of the re-injured group), and post-RTP hour (*n* = 40; *n* = 8 of the re-injured group). Analyses showed that MD values at 24–48 h and asymptomatic visits were not statistically different with and without those missing data at the post-RTP visit. Diagnosis of concussion was defined according to the DoD Consensus as previously described (17).

MRI protocols/processing have been previously described (10). Scans were acquired on Siemens MAGNETOM 3T Tim Trio or Prisma scanners at Virginia Tech, University of North Carolina at Chapel Hill, and University of California, Los Angeles. A single-shot echo planar imaging sequence with a twice refocused spin echo was collected: 30 directions at a *b* value of 1,000 s/mm^2^, 8 b_0_ images, TE/TR = 98/7,900 ms, FOV = 243 mm, matrix size = 90 × 90, 60 slices, and voxel size = 2.7 mm isotropic. Images were denoised and then corrected for motion, eddy current artifacts, and geometric distortion using FSL (18).

Image processing included pre-processing followed by computation of DTI metrics. Using the local principal component analysis (LPCA) approach, diffusion-weighted images were first denoised (19). With a pair of reverse-phase–encoded b0 images as reference, diffusion-weighted images were then corrected for motion, eddy current artifacts, and static-field geometric distortion using the eddy_openmp command provided in the FMRIB Software Library [FSL; (18)]. The transformation matrices output from the eddy_openmp command were used to rotate the corresponding diffusion-weighting directions to match the rotation of the brain image during the motion-correction procedure using an in-house Matlab script. A linear fitting algorithm (using an FSL drift command) enabled DTI metrics to be computed voxelwise. Images were then transformed to the standard Montreal Neurological Institute (MNI) space using Advanced Neuroimaging Tools (ANTs) non-linear registration (20). FSL's Tract-Based Spatial Statistics [TBSS; (21)] was used to perform voxelwise analysis. Diffusions metrics were projected onto a common white matter skeleton. Nonparametric permutation-based statistics used in TBSS (i.e., the randomize command) were used for voxelwise statistical analysis. A threshold-free cluster enhancement (22) and 5,000 permutations (23) were used in this study. Voxels were deemed significant if *p* < 0.05 after being adjusted for multiple comparisons by controlling family-wise error rate (FWER) within the white matter skeleton. The regression models used in TBSS controlled for pertinent demographic factors (i.e., age and sex), as well as site and scanner differences, as previously described (10).

### *Post-hoc* Region-of-Interest Analysis

**Figure 1:**
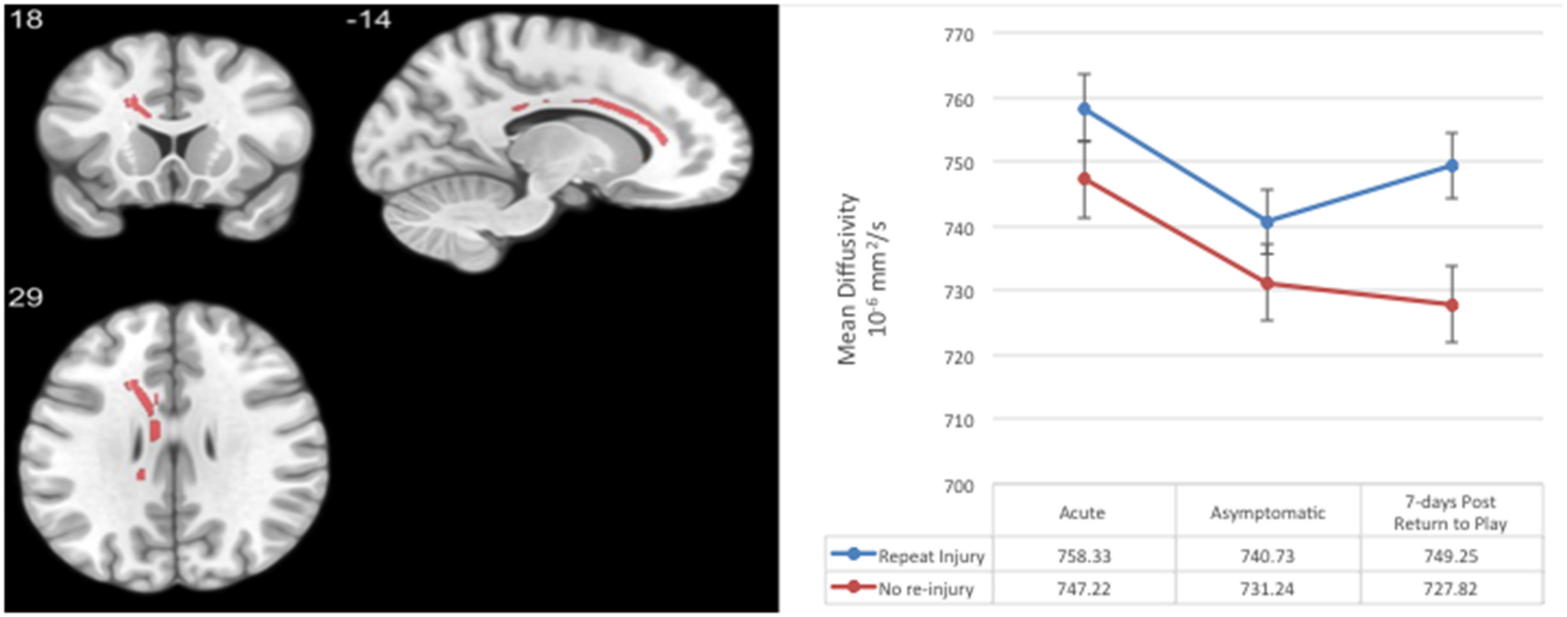
Region-of-interest (ROI) consisting of voxels with significantly elevated mean diffusivity in concussed relative to contact and non-contact control athletes across all post-injury visits in a prior analysis (10). Average mean diffusivity at the acute, asymptomatic, and 7-days post-return-to-play post-concussion visits in concussed athletes with and without a subsequently repeat injury. Error bars represent standard deviation.

Each of the TBSS regression analyses produced significant white matter voxels, as reported by Wu et al. (10). An intersection mask of significant voxels at each visit was created, consisting of 512 voxels in the genu and body of the corpus callosum (Figure 1). Average MD in this region of interest (ROI) was used in subsequent analyses.

### Statistical Analysis

Statistical analyses were conducted using SPSS (v24). Independent sample *t* tests compared continuous demographic characteristics, symptom reporting, and diffusion metrics between concussed athletes with and without subsequent re-injury. Chi-squared and Fisher's exact tests compared categorical or binary demographic characteristics and injury-related parameters (e.g., LOC) between concussed with and without subsequent re-injury. Binary logistic regressions assessed likelihood of repeat injury with the average MD at each post-injury visit in the *a priori* ROI entered as the predictor variable. Bias-corrected and accelerated (BCa) bootstrap intervals were computed for logistic regression analyses (5,000 samples). An alpha of 0.05 was used to determine statistical significance, with 0.10 for non-significant trends. Correction for multiple comparisons was not performed due to the exploratory nature and limited power of the analyses. Athletes in their last year of athletic eligibility or who did not have year of participation listed (*n* = 11) and experienced a secondary injury beyond 1 year (*n* = 3) were excluded, as the monitoring period for re-injury was 1 year (i.e., extending into the subsequent athletic season).

## Results

Sixty-nine out of 82 initially injured athletes met exclusion criteria; 11 experienced a second concussion over the 1-year period (365 days; Table 1). Those who were re-injured within a 1-year period had significantly higher single word reading standard scores on the Wechsler Test of Adult Reading, *t* = 2.03, *p* = 0.046. No other baseline differences were observed (Table 1; *p*s > 0.05). Groups did not significantly differ in their time to asymptomatic or RTP (*p*s > 0.05).

**Table 1:** Demographics, medical history, and injury characteristics of not re-injured and re-injured groups.

	**Total sample (*****N*** **=** **69)**		**Concussed athletes without re-injury (*****n*** **=** **58)**		**Concussed athletes with re-injury (*****n*** **=** **11)**	
	**Mean/*N***	**SD/%**	**Mean/*N***	**SD/%**	**Mean/*N***	**SD/%**
Age	19.65	1.12	19.63	1.14	19.73	1.01
Sex (men)	57	82.60%	48	82.80%	9	81.20%
Race						
*White*	35	50.70%	30	51.70%	5	45.50%
*African American*	27	39.10%	22	37.90%	5	45.50%
*Biracial*	4	5.80%	3	5.20%	1	9.10%
*Hawaiian/ Pacific Islander*	2	2.90%	2	3.40%	0	0.00%
*Unknown/No response*	1	1.40%	1	1.70%	0	0.00%
Handedness (right)	58	84.10%	48	82.80%	10	90.90%
Sport						
*Football*	41	59.40%	32	55.20%	9	81.80%
*Lacrosse*	8	11.60%	8	13.80%	0	0.00%
*Soccer*	20	29.00%	18	31.00%	2	18.20%
*Not listed*	0	0.00%	0	0.00%	1	6.70%
Years participating	10.46	3.72	10.78	3.76	8.82	3.25
Weight	201.19	53.970	199.02	56.24	212.64	40.04
Height	71.730	3.5900	71.53	3.66	72.77	3.14
Previous concussion						
*0*	35.000	51.00%	31	53.40%	4	36.40%
*1*	25.000	36.20%	18	31.00%	7	63.60%
*2 or more*	9.000	13.00%	9	15.50%	0	0.00%
GPA	2.84	0.52	2.86	0.55	2.74	0.418
WTAR Word Reading SS^*^	106.17	14.24	104.7	14.33	114.40	10.17
Headache/migraine disorder	5	7.20%	3	5.20%	2	18.20%
Seizure disorder	1	1.40%	1	1.70%	0	0.00%
Sleep disorder	1	1.40%	1	1.70%	0	0.00%
Diabetes	2	2.90%	1	1.70%	1	9.10%
Hearing problems	2	2.90%	1	1.70%	1	9.10%
ADHD	12	17.40%	9	15.50%	3	27.30%
LD	2	2.90%	1	1.70%	1	9.10%
Depression	3	4.30%	2	3.40%	1	9.10%
Bipolar disorder	1	1.40%	1	1.70%	0	0.00%
Unspecific psychiatric disorder	1	1.40%	1	1.70%	0	0.00%
Initial injury situation						
*Competition*	17	24.60%	15	25.90%	2	18.20%
*Practice/training*	52	75.40%	43	74.10%	9	81.80%
LOC	3	4.30%	1	1.70%	2	18.20%
PTA	9	14.30%	6	11.50%	3	27.30%
RGA	9	14.30%	4	7.10%	2	18.20%
Re-injured	11	15.90%	^*^	^*^	^*^	^*^
Time to asymptomatic	8.96	6.47	8.74	6.63	10.05	5.78
Symptom-free wait days	9.00	11.17	9.28	12.16	7.86	5.64
Acute time point MD	749.31	30.36	747.22	31.45	758.33	24.49
Asymptomatic MD	733.06	39.47	731.24	35.01	740.73	56.25
Seven days Post-RTP MD^*^	732.11	27.58	727.82	25.3	749.25	31.36
Number of acute symptoms	10.67	6.14	10.27	6.23	12.4	5.74
Total acute symptom score	25.31	21.14	23.80	20.64	32.0	23.1

* *Statistically significant differences at the p <0.05 level between the re-injured and not re-injured groups using independent t test, chi-squared test, and Fisher's exact test*.

*GPA, grade point average; WTAR, Wechsler Test of Adult Reading; ADHD, attention deficit hyperactivity disorder; LD, learning disorder; LOC, loss of consciousness; PTA, posttraumatic amnesia; RGA, retrograde amnesia; MD, mean diffusivity; RTP, return to play*.

The average duration from first to second injury was 212 days (*SD* = 108.54; median = 214, range = 42–362). Mean MD at 7 days post-RTP was significantly higher in those athletes who went on to sustain repeat concussion than those athletes who did not (*t* = 2.04, *p* = 0.048, *d* = 0.75; Figure 1). Significant differences or trends in mean MD were not observed at other time points (*p*s > 0.10). In this underpowered sample, a binary logistic regression revealed that the relationship between MD at 7 days post-RTP and occurrence of a secondary injury within 1 year approached significance [Wald $\chi_{\left(1\right)}^{2}$ = 3.58, *p* = 0.058, *B* = 0.03, SE = 0.017; OR = 1.03, CI = 0.99, 1.07]. A similar estimate, *B* = 0.03 (BCa CI = −0.01, 0.15), SE = 0.24 (bias = 0.017), and trend association, *p* = 0.071, was observed between MD at 7 days post-RTP and occurrence of a secondary injury when BCa bootstrap intervals were calculated. A test of this model (i.e., including MD at 7 days post-RTP), as compared to the model with only the intercept, was significant, $\chi_{\left(1\right)}^{2}$ = 4.17, *p* = 0.04.

## Discussion

The current study adds to the growing literature on diffusion MRI markers of acute concussion and recovery, while also exploring the association between longitudinal DTI metrics and repeat concussion in collegiate athletes within a 1-year time frame. Our design enabled us to compare the level and trajectory of MD within 48 h of injury, the point of clinical recovery, and 1 week after RTP in a group of athletes who went on to sustain repeat concussion within a year of the initial injury and a group who did not. Both groups showed a similar trajectory of reduction in MD from 48 h post-injury to the point at which they were deemed clinically asymptomatic, suggesting that MD may be a marker of neurobiological recovery. Athletes who were subsequently re-injured within 1 year of initial injury and those who were not, however, showed a divergent pattern of MD 7 days post-RTP. The repeat concussion group exhibited an increase in MD at 7 days post-RTP, while those not re-injured had a continuous, linear decline in MD even after returning to play. These preliminary findings taken together raise the hypothesis that a subset of athletes may experience a perturbation in white matter integrity that is associated with a more prolonged period of risk for repeat injury or overall increased susceptibility (12–14).

The large-scale and prospective design of the CARE Consortium provided the unique ability to examine the relationship between microstructural white matter changes following sport-related concussion (i.e., elevated MD) and a variety of outcomes, including increased likelihood of re-injury, in an attempt to better characterize the clinical meaningfulness of persistent signal of DTI (1). While those not re-injured had a continuous, linear decline in MD even after returning to play, athletes who experienced a subsequent concussion within 1 year exhibited an increase in MD at 7 days post-RTP. This re-elevation of MD at 7 days post-RTP is particularly interesting in the context of a prior study of 15 non-concussed collegiate football players and five matched controls, which reported statistically significant pre- to post-season signal differences in diffusion (i.e., increased FA and decreased trace) that returned to baseline levels (i.e., not statistically different from pre-season) 6 months after the season's end and a period of no-contact rest (24).

Group differences or pre- to post-injury within-subject changes in these metrics following sport-related concussion can generally indicate alterations in white matter structure, and preclinical/experimental studies have reported on associations between FA and axial diffusivity with axonal damage (25–27). However, clinical interpretation of post-injury differences on DTI metrics in human subjects can be challenging due to the non-specific source of diffusion signal that reflects a summary measure of collective diffusion in multiple tissue compartments within a given voxel being measured (28, 29). As a result, the source of the signal is non-specific and could represent a number of intra- or extra-cellular processes, such as extracellular edema, axonal swelling, demyelination, etc. (30). Due to this, identifying the physiological mechanism underlying this potential vulnerability is not clear at this time.

It has been suggested that persistent signal reflects a period in which the brain has not yet fully physiologically recovered. The current data, although preliminary, support the hypothesis that persistent physiological effects of sport-related concussion (i.e., those that extend beyond clinical recovery) may reflect a window of cerebral vulnerability or are otherwise associated with an increased susceptibility following concussion in which athletes that have returned to play are at increased risk for a secondary injury. Findings from the original NCAA study investigating the natural course of sport-related concussion between 1999 and 2001 reported that approximately 92.0% of repeat concussions within the same season occurred within 10 days of initial injury, also supporting the notion of an ongoing window of cerebral vulnerability during physiological recovery (15). Relatedly, a separate study of 635 concussed high school and collegiate athletes observed that those with a shorter symptom free waiting period (i.e., faster RTP with decreased time for physiological recovery) were significantly more likely to experience a secondary SRC within the same year (31).

Limitations of the study include possible reduced power to detect true effects due to our small sample of athletes with repeat concussion, which is expected given the low base rates of re-injury within the same year (16). Due to the low base rate of repeat injuries, a conservative approach for multiple comparison correction was taken. Additionally, missingness of neuroimaging data at different time points did occur for select subjects; however, sensitivity analyses suggested that MD values at 24–48 h and asymptomatic visits were not statistically different with and without those missing data at the post-RTP visit. These findings require replication and future studies should aim to identify the mechanism underlying the association between persistent signal on DTI (i.e., elevated MD) and the potential persistent physiological risk of re-injury.

These preliminary findings raise the hypothesis that a subset of athletes may experience a perturbation in white matter integrity that is associated with a more prolonged period of risk for repeat injury or overall increased susceptibility. The current findings require replication and future studies should aim to identify the mechanism underlying the association between persistent signal on DTI (i.e., elevated MD) and the potential persistent physiological risk of re-injury.

## Data Availability Statement

These data will be publicly available through the Federal Interagency Traumatic Brain Injury Research (FITBIR) platform (https://fitbir.nih.gov/content/access-data).

## Ethics Statement

The studies involving human participants were reviewed and approved by The Medical College of Wisconsin Institutional Review Board. The patients/participants provided their written informed consent to participate in this study.

## Author Contributions

BB, MM, and TBM designed and conceptualized study, analyzed data, interpreted data, and drafted the manuscript for intellectual content. Y-CW major role in the acquisition of data, interpreted data, and drafted the manuscript for intellectual content. SM, AS, KK, AN, CG, JG, KG, JM, and SD major role in the acquisition of data and revised the manuscript for intellectual content. SB and TWM designed and conceptualized study, major role in the acquisition of data, and revised the manuscript for intellectual content. All authors approved the final manuscript as submitted and agree to be accountable for all aspects of the work.

### Conflict of Interest

The authors declare that the research was conducted in the absence of any commercial or financial relationships that could be construed as a potential conflict of interest.

## Abbreviations

ANTs: advanced neuroimaging tools
DTI: diffusion tensor imaging
FSL: functional MRI of the brain software library
MD: mean diffusivity
MRI: magnetic resonance imaging
ROI: region of interest
TBSS: tract-based spatial statistics
WTAR: Wechsler Test of Adult Reading.
